# Effectiveness of Integrated Health Systems in Africa: A Systematic Review

**DOI:** 10.3390/medicina56060271

**Published:** 2020-05-29

**Authors:** Irene G. Ampomah, Bunmi S. Malau-Aduli, Aduli E.O. Malau-Aduli, Theophilus I. Emeto

**Affiliations:** 1 College of Public Health, Medical and Veterinary Sciences, James Cook University, Townsville QLD 4811, Australia; irene.ampomah@my.jcu.edu.au (I.G.A.); aduli.malauaduli@jcu.edu.au (A.E.O.M.-A.); 2 College of Medicine and Dentistry, James Cook University, Townsville QLD 4811, Australia; bunmi.malauaduli@jcu.edu.au

**Keywords:** traditional medicine, integration, review, public health, health system

## Abstract

*Background and objective*: Traditional medicine (TM) was integrated into health systems in Africa due to its importance within the health delivery setup in fostering increased health care accessibility through safe practices. However, the quality of integrated health systems in Africa has not been assessed since its implementation. The objective of this paper was to extensively and systematically review the effectiveness of integrated health systems in Africa. *Materials and Methods*: A systematic literature search was conducted from October, 2019 to March, 2020 using Ovid Medline, Scopus, Emcare, Web of Science, Cumulative Index to Nursing and Allied Health (CINAHL), and Google Scholar, in order to retrieve original articles evaluating the integration of TM into health systems in Africa. A quality assessment of relevant articles was also carried out using the Quality Assessment Tool for Studies with Diverse Designs (QATDSS) critical appraisal tool. *Results*: The results indicated that the formulation and execution of health policies were the main measures taken to integrate TM into health systems in Africa. The review also highlighted relatively low levels of awareness, usage, satisfaction, and acceptance of integrated health systems among the populace. Knowledge about the existence of an integrated system varied among study participants, while satisfaction and acceptance were low among orthodox medicine practitioners. Health service users’ satisfaction and acceptance of the practice of an integrated health system were high in the countries assessed. *Conclusion*: The review concluded that existing health policies in Africa are not working, so the integration of TM has not been successful. It is critical to uncover the barriers in the health system by exploring the perceptions and experiences of stakeholders, in order to develop solutions for better integration of the two health systems.

## 1. Introduction

Traditional medicine (TM) refers to the sum total of all explicable knowledge and practices used in the diagnosis, prevention, and elimination of physical, mental, and social imbalance. TM exclusively relies on practical experience and observations handed down verbally or in writing from generation to generation [1]. TM’s use is increasing in both developed and developing countries. For example, the proportion of residents who have patronised TM at least once in developed countries is 38% in Belgium, 42% in the United States of America, 48% in Australia, 70% in Canada, and 75% in France [2]. TM use is reported to be more popular in Africa, Asia, and Latin America, since 80% of the population continue to depend on TM for their primary health care needs [2,3]. The high frequency of TM use in Africa has been attributed to reasons such as TM being economical, and socially and culturally acceptable within the African setting [4,5,6]. Additionally, TM has proven to be effective in managing and treating tropical maladies and other ailments, such as epilepsy, hypertension, insomnia, ovarian cancer, convulsion, stroke, boils, tuberculosis, infertility, hernia, and malaria, among others [3,6,7]. In Nigeria, for instance, *Rauwolfia vomitoria,* a medicinal herb in the *Apocynaceae* family, is used to manage disease conditions such as insomnia, convulsion, stroke, and hypertension [3]. Traditional birth attendants in South Africa use TM with muscle relaxant characteristics to help with the safe delivery of babies [3]. *Aloe vera*, black seed, black cohosh, and other TM plants are acknowledged for their outstanding health promotion and disease prevention benefits [8]. Studies have reported that TM continues to be an essential ingredient of a number of medications currently used for the management of heart diseases, fevers, pain therapies, and other health problems [3,9]. For instance, artemisinin, a derivative of the medicinal plant *Artemisia annua*, is the origin of a suite of efficient antimalarial drugs [3]. Recognition of the important role that TM plays in health delivery (its high prevalence and medicinal benefits) led to its integration into various health systems around the world, including those of Africa [4].

Integration has been defined as the act of broadening the scope of health delivery through communication, participation, accommodation, and partnership building between biomedical and traditional health systems, while safeguarding indigenous medical knowledge [10]. The sole purpose of an integrated health system is to offer quality health care to the population equally and satisfactorily, while averting unnecessary costs [11]. A booming integrated health system is likely to promote the proper use of indigenous medical knowledge and boost the development of health systems (self-adequacy), particularly in poor-income countries [12]. Asian countries such as Korea, Japan, China, India, Sri Lanka, and Vietnam have successfully integrated TM into their health system [12,13]. The practice of TM in these countries is founded on logical and comprehensive methods, as well as clinical experiences [13]. For example, in China, biomedical doctors are trained in the field of TM practice in order to support competent TM practitioners in health centers, embrace treatment processes based on TM principles, gain understanding from experienced TM practitioners, and study the curative effect of TM treatment with the diagnosis and model of the orthodox health system. This approach not only boosted the confidence of biomedical doctors in the field of TM, but also improved and sustained collaboration between the two health systems [13]. The Chinese success story in the field of integrated health is also illustrated by a rise in registered Chinese TM practitioners in the United States of America [14]. India has also boosted its integrated system through the incorporation of the repayment of medical costs incurred by utilizing TM products and services. Thereby, Indians who work in the public sector are reimbursed money spent on TM products and services [15]. 

African countries have also made efforts to integrate TM into formal health systems [4]. Such countries include Benin, Burkina Faso, Cameroon, Cote d’Ivoire, Equatorial Guinea, Ghana, Guinea, Mali, Mozambique, Niger, Nigeria, and the Republic of Congo, among others [10,16]. For example, Ghana instituted a council in the year 2010 to regulate the activities of TM practice and, in 2012, registered TM practitioners were allowed to practice medicine [17]. Similarly, Nigeria also formulated the National Policy on the TM Code of Ethics and the creation of national and state TM boards to oversee the practice, and boost partnership and research in the field of TM [18]. Most African countries ascribe to a parallel/inclusive model of health system integration. A parallel or inclusive model is a system where TM and biomedical health care are separate elements of the health system, but both are expected to interact and work jointly to deliver quality health services to clients [10,16]. However, the effectiveness of the integration of TM into health systems to offer quality health services to the African populace is unexplored. Therefore, this systematic review presents a comprehensive assessment of published literature on the effectiveness of integrated health systems in Africa. The primary measures of effectiveness in this study are the awareness, usage, satisfaction, and acceptance of an integrated health system among study populations. 

## 2. Materials and Methods

### 2.1. Defining the ‘Integration of TM’

In this systematic review, TM integration refers to any research that has focused on the incorporation of herbal/indigenous medicines, including bonesetters, into health systems. Integrated health systems also include partnership between orthodox and traditional health service providers. The Preferred Reporting Items for Systematic Review and Meta-Analysis (PRISMA) guidelines were adapted for this review [19]. A literature search was performed from October, 2019 to March, 2020 using Ovid Medline, Scopus, Emcare, Cumulative Index to Nursing and Allied Health (CINAHL), Web of Science, and Google Scholar to retrieve original articles on the integration of TM into health systems in Africa. Various keywords or synonyms were used in the search strategy to expand the search term because a number of terms have been used in the literature to refer to the same concept. Although the review is focused on the effectiveness of integrated health systems in Africa, the words ‘effective’ and ‘Africa’ were not included in the search terms to avoid restrictions of the search. Africa was precluded from the search term to avoid excluding articles reporting research conducted in Africa, but without the word ‘Africa’ in their titles. Minor differences existed in the search terms, depending on the type of database and search engine (Supplementary Table S1). 

### 2.2. Eligibility Criteria 

There were no restrictions for the time and type of study, but the study setting was limited to Africa. Articles included in this systematic review are primary studies published in peer-reviewed journals reporting on measures taken to aid TM integration into health systems and the effectiveness of such integrated systems. Studies which referred to TM as herbal/indigenous medicine were selected for the review. The review excluded the following: Systematic reviews, theses, non-English articles, and other articles with animals as the target population.

### 2.3. Selection and Extraction of Data

I.G.A identified relevant articles and T.I.E replicated the search to confirm the search strategy. Uncertainties regarding the included studies were resolved by discussion, until a consensus was reached. The characteristics obtained from included studies were the target population, type of study, methodology, measures taken to integrate TM, summary of findings/results, and effectiveness of TM integration. However, interventions implemented to integrate TM into formal health systems were the key inclusion criteria.

### 2.4. Data Synthesis

The included studies were evaluated based on interventions implemented to integrate TM into formal health systems and key findings in relation to the effectiveness of the integrated health system. It is worth mentioning that the effectiveness was inferred from the findings and conclusions of various studies. This is because the effectiveness of integrated health systems was described, rather than explicitly stated, in the findings. Inference was conducted by evaluating the level of awareness, usage, satisfaction, and acceptance of the integration of TM among target populations. Awareness was inferred based on self-reported knowledge of the introduction of TM into the health system, while usage was deduced from the interactions between stakeholder groups (orthodox and TM practitioners, and health service users). Satisfaction was deduced on the basis of the fulfillment that participants derived from the system and acceptance was gleaned based on the value that participants placed on their role and the role of other stakeholders in the integration agenda. With quantitative studies, percentage scores correlating to inferred knowledge, usage, satisfaction, and acceptance which were less than or equal to 39% were deemed low [20], while values of 40% and above were assumed to be moderate/positive indicators [17]. Similar inferences were conducted for qualitative studies and representative quotes were used to support the inferences drawn. 

### 2.5. Quality of Methods Assessment

The Quality Assessment Tool for Studies with Diverse Designs (QATSDD) developed by Sirriyeh et al., was used to evaluate the quality of included studies [21]. The tool was adopted because it was applicable in all instances, since the review identified and included all types of studies. The tool is made up of 16 criteria and all the criteria were applicable to the mixed methods study, while qualitative and quantitative studies were assessed using fourteen 14 of these criteria. Each criterion on the list was allotted a score of zero to three, where 0 represents ‘criterion not mentioned at all’, 1 represents ‘very slightly mentioned’, 2 represents ‘criterion moderately stated’, and 3 represents a ‘vivid explanation’ of the criterion. In view of this, the highest mark for the mixed methods study was 48 (16 × 3) and 42 (14 × 3) was the highest mark for the qualitative and quantitative studies, respectively. The total scores of included studies were further converted to percentages. For example, a mixed methods study with a score 48 out of 48 equates to 100% (48/48 × 100 = 100). The percentage scores were further categorised into three groups such that an article was considered excellent if the percentage score was 80 and above, good if scores ranged between 50% and 79%, and low if the score was below 50%.

## 3. Results 

**Figure 1:**
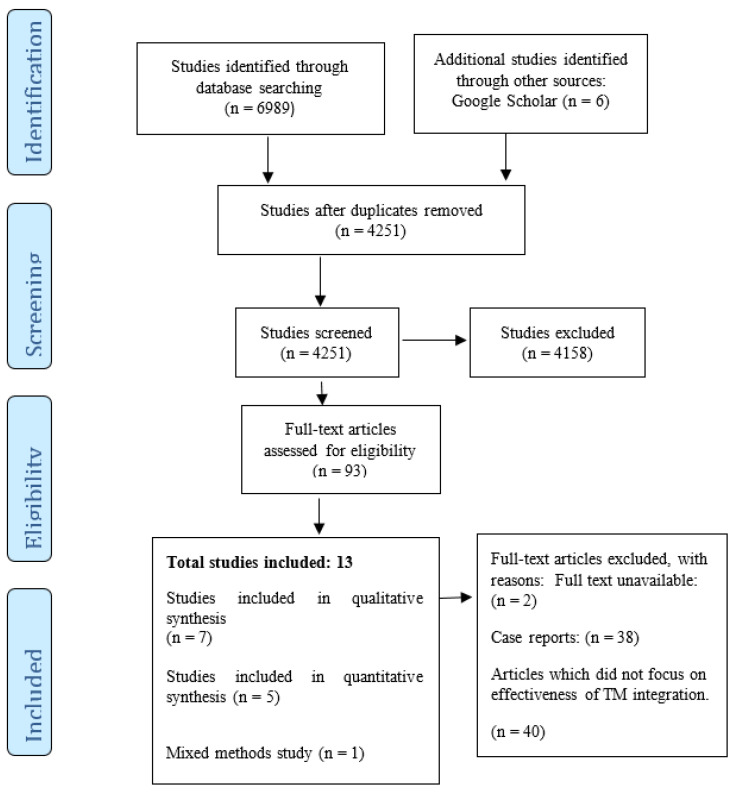
PRISMA (The Preferred Reporting Items for Systematic Review and Meta-Analysis) flow diagram of included studies [19].

In total, 6995 articles were identified from five databases and the Google Scholar search. However, 2744 duplicates were removed, leaving 4251 articles. A total of 93 articles were retained after title and abstract screening. Thirteen out of the 93 articles met all of the inclusion criteria after full text screening (see Figure 1).

### 3.1. Distribution of Reviewed Articles 

**Figure 2:**
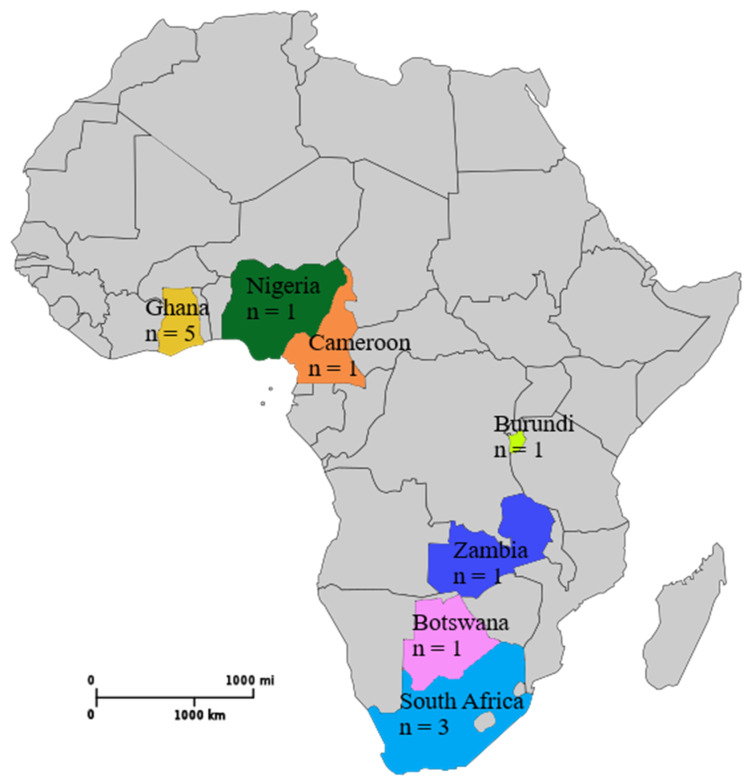
Map of Africa displaying the number of studies originating from various countries taken from Wikimedia Commons [32]. Accessed: 16 December 2019.

Thirteen articles were included in the review. Five (38.5%) of the articles originated from Ghana [12,17,22,23,24]. Three studies (23.1%) were conducted in South Africa [25,26,27]. The remaining five studies were carried out in Burundi [28], Botswana [20], Cameroon [29], Nigeria [30], and Zambia [31]. The reviewed studies were conducted over a period of thirteen years. The earliest study [31] was conducted in 2006, while the latest studies were carried out in 2019 [24,27]. Figure 2 shows the number of included studies from different countries in Africa.

### 3.2. Characteristics of Reviewed Articles 

Of the 13 included studies (Figure 1), seven were qualitative and five were quantitative studies. Only one was a mixed methods study. Two of the qualitative studies used a phenomenology approach (qualitative research which emphasizes similarities in lived experiences within a specific group of people) to achieve the study objectives [22,27], while one study [12] used an inductive reduction approach. Ethnography (qualitative research in which the researcher defines and interprets common and learnt forms of behavior, values, beliefs, and language of a culture-sharing group) was employed as research design for the qualitative aspect of the mixed methods study [28]. However, three of the qualitative studies did not state the research design used [23,25,26]. All quantitative studies used a cross-sectional design in achieving the study objectives [17,20,29,30,31]. 

As shown in Table 1, four studies targeted health practitioners and patients as study participants [12,22,25,28]. Two studies only focused on health practitioners [24,31]. Two other studies solely targeted TM practitioners [27,30], while another two focused on orthodox medicine practitioners (OMPs) [20,26]. One study assessed both TM practitioners and community members [29]. Another study also considered people interested in TM research as the target population [23]. The last reviewed article only targeted patients [17]. More focus was placed on TM practitioners, as nine of the included studies sampled TM health providers as study participants [12,22,24,25,27,28,29,30,31]. Only one study included key informants, consisting of the Faculty of Pharmacy (FOP), Ghana Federation of Traditional Medicine Practitioners (GFTMP), Medical Herbalist Association (MHA), Hospital Management (HM), and Pharmaceutical Directorate of Ministry of Health (PDMH), as part of the study group [22]. 

The sample size of quantitative articles ranged from 60 [20] to 6690 [28] participants. The most common sampling technique employed by quantitative studies was the convenient sampling procedure [20,29]. Other techniques used were systematic [17] and stratified sampling [28]. Two studies [30,31] failed to state the sampling techniques employed in choosing participants for the study. For qualitative studies, it ranged from 27 [27] to 37 participants [25]. The purposive sampling technique was widely used among qualitative studies [22,23,25,26]. Another sampling approach used was snowballing [12,28]. However, one study combined both snowballing and purposive sampling techniques in participant selection [24]. One qualitative study failed to mention the type of sampling technique employed in choosing participants for the study [27]. Generally, all reviewed articles assessed the perception, knowledge, acceptability, and satisfaction of study participants in relation to TM integration. The review inferred the effectiveness of integrated health systems on the premise of participants’ awareness, usage, satisfaction, and acceptability of TM integration into formal health systems. Twelve of the studies disclosed that integrated health systems in Africa were not effective [12,20,22,23,24,25,26,27,28,29,30,31]. Ineffective TM integration was appraised on the basis of a low level of participants’ knowledge about TM integration into health systems, minimal interaction among stakeholders within the integrated system, inadequate satisfaction derived from accessing or practicing in the system, and power imbalance within the integrated systems. Only a single study reported moderate/good indicators for the integrated health system among study participants [17]. 

### 3.3. Evaluation of the Effectiveness of Integrated Health Systems in Africa 

#### 3.3.1. Interventions Implemented to Aid the Integration of TM into Health Systems in Africa

The official practice of TM started in the early 1980’s [29]. This means that the practice of an integrated health system in Africa has been in operation for approximately 39 years (Table 2). All reviewed articles acknowledged that TM integration was initiated through the formulation and execution of health policies [12,17,20,22,23,24,25,26,27,28,29,30,31]. Countries such as Burundi, Ghana, and South Africa went a step further by implementing other measures to facilitate the integration process. For example, in Burundi, the association of TM practitioners was formed and an integrative medicine unit was created in the Ministry of Health [28]. In Ghana, the government instituted the Traditional Medicine Practice Council (TMPC), established a TM directorate in the Ministry of Health, and introduced TM into the tertiary education system [12,17,23]. In South Africa, a council was created to oversee the activities of TM practitioners [27]. 

#### 3.3.2. Awareness as a Measure of an Effective Integrated Health System in Africa

Varying levels of awareness were observed among review articles. Seven studies reported a low level of awareness about the existence of integrated health systems in their countries [20,22,24,26,29,30,31]. For example, Kaboru, Falkenberg [31] recounted that a lower proportion (24%) of study participants claimed knowledge of the practice of an integrated health system in Zambia. A similar result was observed in the work of Madiba [20], where only 18.6% of participants knew of the integrated health system in Botswana. The knowledge gap was more profound in the area of availability of TM policy which regulates the activities of practitioners. However, six studies reported a moderate level of awareness among study participants [12,17,23,25,27,28]. For instance, a study conducted in Ghana reported that 42.2% of participants knew about the presence of TM units in the study sites [17] and 91% of orthodox medicine practitioners in Burundi were also aware of the integrated health system [28]. Participants attributed their source of knowledge to the interaction between traditional and orthodox health practitioners, but also admitted that integration was weak (Table 2). 

#### 3.3.3. Usage as a Measure of an Effective Integrated Health System in Africa

The usage of an integrated health system was inferred as the interaction between stakeholders (orthodox medicine practitioners, TM practitioners, and health care users) within the health system. A total of ten articles reported a low usage of the health system among study participants [12,20,22,23,24,26,27,28,29,30]. The usage of an integrated system was particularly low among orthodox practitioners, as only 27% of participants had ever collaborated with TM providers and 10% were willing to refer clients to TM providers [20]. The under-utilization of integrated health systems in Africa was attributed to the absence of protocol to publicize the integrated health system [22], TM products, and services not included in national health cover [23] and an unwillingness of orthodox practitioners to embrace the incorporation of TM practice into formal health systems [20,24]. Generally, inadequate evidence-based research to support TM practice was cited by orthodox practitioners as the reason for their resistance. Nonetheless, three studies reported a moderate usage of integrated health systems [17,25,31]. A Ghanaian study reported that 42.2% of participants patronized health services offered at the TM units in the study settings [17]. However, 13% of the participants stated that usage of the integrated system could increase through positive recommendations from orthodox practitioners. (Table 2). A study conducted in Zambia reported a moderate level of usage, as 53% of TM practitioners interacted with the formal health system by advising clients to access certain biomedical services, such as laboratory services, before they (TM practitioners) commenced treatment [31]. Usage was reported to be moderate in South Africa, as health service users patronized both health systems simultaneously or at different time periods, depending on the efficacy of treatment. For example, a participant stated, “*We commenced treatment at orthodox health care, then progressed to TM providers but accessing TM worsened the ailment. So we stopped and returned to orthodox health care*” [25].

#### 3.3.4. Satisfaction as a Measure of an Effective Integrated Health System in Africa

Satisfaction was determined by the level of fulfilment that participants derived from integrated health systems. Ten reviewed articles reported a low level of satisfaction among participants [12,20,23,24,25,26,27,28,29,30]. Satisfaction was recounted to be minimal because 70% of participants were not pleased with TM practice [20]. This low satisfaction was mainly due to the failure of policy makers to enact and implement policies to promulgate the integration process, weak communication within the referral system between health care providers, and lopsided power relations within integrated health systems. However, one study reported that 53% of study participants were satisfied with the integrated health system because their preference for TM had increased due to its operation in health centres [17]. Another study reported varying levels of satisfaction among participants. The variation ranged from ‘being satisfied with the system’ (e.g.,”*The collaboration is very strong. There are sometimes intra referrals from TM units to orthodox and vice versa. Clients go to Out Patients Department, then to TM unit, we check vital statistics, then clients would be examined and recommended to do some laboratory test when necessary. We ensure constancy for the sake of report”—*TM practitioner) to ‘not satisfied with the system’ (e.g., “*We have not admitted any client who patronise TM and orthodox medicine. I don’t consider it as integration, because we are not working jointly with them”—*orthodox medicine practitioner) [22]. 

#### 3.3.5. Acceptance as a Measure of an Effective Integrated Health System in Africa

Acceptance was closely linked to the satisfaction of participants. While satisfaction evaluated the fulfilment that people derived from the integrated system, acceptance was deduced based on the value that participants placed on their role and the role of other stakeholders in the integration process. Eight of the reviewed pieces of literature indicated that the acceptance of integrated health systems in Africa was low among orthodox medicine practitioners [12,20,22,24,25,26,28,29]. For example, Falisse et al. [28] found that, although the majority of orthodox medicine practitioners in Burundi (91%) were aware of the integrated system, only 19% supported formal integration [28]. Related results were disclosed by a Ghanaian study, where orthodox medicine practitioners were highly unprepared to refer clients to TM practitioners [12]. Likewise, orthodox practitioners in South Africa frowned at the integration process because they (OMPs) perceived TM practice to be an obstacle to successful management of clients’ health [25]. Differing levels of acceptance were observed among TM practitioners. A Nigerian study [30] reported a high level (64%) of acceptance among TM practitioners at Mushin, Lagos, and the findings of Maluleka and Ngoepe [27] identified a contradictory report, as acceptance of the integrated system was low among TM practitioners in South Africa. This was because they (TM practitioners) experienced marginalization in the integrated system. One study found that perceived acceptance was reported to be high among both health practitioners in Ndola and Kabwe, in the Copper belt and Central provinces of Zambia. This was due to the fact that 77% of orthodox health providers and 97% of TM practitioners felt that there was a potential prospect for them to learn from each other in order to work effectively [31]. Overall, the practice of an integrated health system was popular among health service users. Two studies reported a high acceptance of an integrated system among service users in Ghana [12,17]. It also emerged that the practice of an integrated health system was highly favorable among scholars involved in TM research [23]. 

### 3.4. Assessment of the Methodological Quality 

As indicated in Table 3, the included studies were of a good methodological quality and had QATSDD scores ranging from 57% to 86%. One study scored above 80%, and was thus classified as having an excellent methodological quality. Five studies scored above 70% and none of the studies were below 50%. The average methodological quality score of the included studies was 69%, which equates to an acceptable standard based on the criteria of the QATSDD assessment tool. Overall, the included studies provided detailed information about sampling, data collection methods, data analysis, and the strengths and limitations of the study. 

## 4. Discussion 

TM plays a significant role in health delivery, which has led to its integration into health systems of various African countries [33]. The rationale behind the integration of TM is to widen the scope of health delivery and improve health seeking behavior among populations [4]. Published research has shown that integration can only be successful if contextual factors, such as the health system architecture, socio-cultural characteristics, and views of stakeholders, are cautiously considered in the integration process [34]. Other important determinants include those directly related to health practitioners, service users, and the broader socio-political structure within which health systems operate [34]. Studies have also shown that the level of effectiveness of an integrated health system mostly relies on the nature of the relationship that exists between all stakeholders in the health sector [34,35].

This systematic review was therefore conducted to assess the effectiveness of integrated health systems in Africa. The measure of effectiveness was based on the awareness, usage, satisfaction, and acceptance of integrated systems. Reviewed articles reported that integrated health systems in Africa were ineffective. Knowledge about the existence of integrated systems was low in most countries and health service users favored the integration of TM into formal health systems. This result is mirrored in the works of Ben-Arye, Karkabi [36] and Jong, van de Vijver [37], where service users agreed that orthodox practitioners should guide clients to choose appropriate TM and refer them to qualified and experienced TM practitioners when the need arises. However, the review identified that the majority of users were uninformed about the official practice of TM in health centers, accounting for the low usage of integrated systems in Africa. Ignorance of service users was attributed to the absence of explicit protocols/documents clearly describing the concept of integration, as well as sensitizing the people about the integration process [22]. However, whilst health professionals, particularly orthodox medicine practitioners, were conversant with the practice of integrated health, their disposition towards the system was poor [28]. Sewitch, Cepoiu [38] confirmed the finding that doctors have an unfavorable attitude towards TM prescription and the referral of clients to TM practitioners. Shallow knowledge about the practice of integrated systems in Africa might be a factor contributing to the undesirably low patronage integrated health care. 

The review established that the patronage of integrated health systems was unimpressive due to ineffective communication as a result of a non-functional referral system, the absence of an official document to promote the integrated system, the non-inclusion of TM products and services in national health cover, and skewed power relations within the integrated system [12,22,23,27,28,30]. The unsuccessful collaboration between the two health systems affected the quality of services offered by the consolidated health delivery unit. Therefore, the performance of integrated systems was reported to be unsatisfactory because health practitioners and service users such as stakeholders expressed dissatisfaction with the state of health systems in Africa. 

Satisfaction is said to be achieved when health practitioners, together with service users/clients, derive fulfilment in practicing and accessing health services [39]. Most health systems in Africa have not achieved this goal. The review indicated low satisfaction among stakeholders. The unsatisfactory state of health systems in Africa mainly stems from inadequately trained TM practitioners, the slow rate of progress of the integration process, and incompetent measures aimed at eliminating charlatan practitioners [23,28]. Conversely, Agyei-Baffour, Kudolo [17] reported a rise in the use of TM among service users/clients because of moderate satisfaction derived from the integrated system. Orthodox medicine practitioners were not satisfied with the integration of TM into formal health systems because they deemed TM practice to be an obstacle to achieving a healthy population [25]. The absence of a common professional language between the two practitioners further deepened the displeasing state of integrated systems. This discovery is closely linked to the findings of Hollenberg [40] in Canada, where differences in terminologies created communication barriers between orthodox and TM practitioners and hindered professional collaboration in integrative health care facilities. This notwithstanding, TM practitioners were somewhat pleased with the system and acknowledged that there was hope for better integration of the two health entities. The review further assessed the acceptance of the practice of integrated systems. 

Acceptance of the practice of integration was low among health practitioners, particularly orthodox health care providers, but high among health service users. This was mirrored in the findings of Falisse, Masino [28], as a relatively low percentage (19%) of orthodox practitioners supported formal integration, while a significant percentage (93%) of clients preferred integration. Likewise, the results of Maluleka and Ngoepe [27] identified that acceptance within the system was low among TM practitioners due to feelings of ostracization. The feeling of exclusion expressed by TM practitioners was a product of a sense of superiority by orthodox providers in the integration process, as well as exclusion of TM in the educational curriculum. Taken together, this review has unraveled key factors responsible for the non-functionality of health systems in Africa. Clearly, for health systems to be effective, more than mere lip service to policy formulations is required, and such policies should include other extraneous factors or interventions. Good health system research is needed to identify specific factors which impede the effective integration of TM into health systems. Future studies should focus on analysing the perceptions and experiences of stakeholders in relation to the integration process within a wider socio-political context. 

### Strengths and Limitations

This review relied upon reported experiences from participants to highlight the state and effectiveness of integrated health systems in Africa. To the best of our knowledge, this is the first article to assess the effectiveness of integrated health systems and factors impeding the integration process in Africa. However, the included studies were not evenly distributed as a majority of the studies were from Ghana [12,17,22,23,24]. The exclusion of grey literature and non-English articles created possible grounds for oversight. Furthermore, the QATSDD appraisal tool largely depends on reviewers’ knowledge. Therefore, there is a potential for bias [21]. There is also the possibility of misclassification and recall biases, since participants had to recollect their experiences with the integrated health system. 

## 5. Conclusions 

In Africa, the main step taken by countries to integrate TM into formal health systems is policy formulation and the creation of TM practice councils. Some countries have managed to establish institutions responsible for assessing the efficacy of TM and introduced TM practice into the tertiary educational system. Existing health policies are not working, so the integration of TM has not been successful. It is critical to uncover the bottlenecks in the health systems by exploring the perceptions and experiences of stakeholders, in order to offer solutions for better integration of the two health systems. 

## Figures and Tables

**Table 1:** Characteristics of studies on the effectiveness of integrated health systems in Africa.

Reference	Study Setting	Target Group/Size	Study Type	Study Design	Aims/Objectives	Sampling Technique	Summary of Findings
Boateng et al. [22]	Ghana: Kumasi South Hospital	1 FOP, 1 PDMH, 1 MHA, 1 GFTMP, and 1 HM patients; 10 (biomedical), 6 (TM clinic), and 2 biomedical practitioners; 2 medical herbalists; 8 nurses (biomedical); 1 nurse (herbal clinic)	Qualitative	Phenomenology	Explored integration of traditional and biomedical health services within Kumasi South hospital	Purposive sampling	Stakeholders had a varying understanding concerning integration. This was a result of the non-existence of a well-defined protocol for guiding the integration process.
Agbor and Naidoo [29]	Cameroon: Bui division	21 traditional medicine practitioners (TMPs) and 52 inhabitants of Bui who sought treatment in hospitals or from traditional healers for oral problems	Quantitative	Cross-sectional	Evaluated the knowledge and practices of TMPs	Convenience sampling	TMPs play a significant role in health delivery in Bui Province, yet their integration into the main health system is weak due to inadequate professional education on the part of TMPs.
Agyei-Baffour et al. [17]	Ghana: Kumasi	Patients from the 3 facilities with TM units in the Ashanti region: 212 from Kumasi South hospital, 112 from Tafo hospital, and 174 from Suntreso government hospital	Quantitative	Cross-sectional	Determine client perception, disclosure, and acceptability of integrating herbal medicine into mainstream healthcare in Kumasi	Systematic sampling	The practice of an integrated health system was reported to be feasible as the satisfaction and acceptance levels among participants were high.
Ahenkan et al. [24]	Ghana: Wenchi municipality	35 orthodox and TMPs	Qualitative	Relational analysis	Aimed to bridge the existing information and knowledge gaps on the integration of two districts and competing health systems.	Snowballing/purposive	The integrated health system in Ghana was said to be inefficient because regulatory mechanisms were unknown to health practitioners. This, according to the study, was due to inefficient policy implementation, and the slow pace of executing regulations governing TM practice in Ghana.
Appiah et al. [23]	Ghana	22 KNUST 1 center for plant medicine research 2 piloted hospitals	Qualitative	Not stated	Identified the strengths and weaknesses of the integration of traditional medicine into existing biomedical practice in Ghana.	Purposive sampling	Integrated health system in Ghana was recounted to be ineffective due to inadequate publicity, documentation, and effort from policy makers.
Awodele et al. [30]	Nigeria: Mushin; Lagos	170 traditional medicine practitioners (TMPs)	Quantitative	Cross-sectional	Explored TMPs’ disposition towards the integration of traditional medicine.	Not stated	The willingness of TMPs to improve their practice was a factor identified to boost integration, while a lack of regulatory protocol undermined integration.
Campbell-Hall et al. [25]	South Africa: KwaZulu-Natal	15 formal health practitioners 1 NGO worker 6 TMPs 15 health users	Qualitative	Not stated	Explored perceptions of service users and providers of the current interactions and mechanisms for increasing collaboration between formal health practitioners and TMPs.	Purposive sampling	Willingness of TMPs was a positive indicator of collaboration. Yet, they felt unappreciated in the integrated system. TMPs were ready to learn the modern style of providing care to users to maximize their value in the integrated process.
Falisse et al. [28]	Burundi	12 indigenous healers 36 biomedical professionals 6690 health care users	Mixed method	Ethnography	Sought to advance the debate on the possibility and usefulness of integration within the socio-politically unstable setting of Burundi.	Stratified sampling and snowballing	The nature of the integrated health system was deemed to be weak, though the idea of integration was popular among participants. The weak nature of integration was attributed to skewed power dynamics in the integrated system.
Gyasi et al. [12]	Ghana: Sekyere south district and Kumasi metropolis	16 health care users 7 TMPs 6 health professionals	Qualitative	Inductive reduction approach	Explored health care users’ and providers’ experiences and attitudes towards the implementation of inter-cultural health care policy in Ghana.	Snowballing	There was a positive attitude towards integration with high awareness level, but inadequate institutional support and regulatory policies were revealed to impede the integration process.
Kaboru et al. [31]	Zambia: Ndola, Kabwe	172 orthodox medicine practitioners 144 TMPs	Quantitative	Cross-sectional	Explored biomedical and TMP experiences of and attitudes towards collaboration.	Not stated	A low level of integration was reported in Zambia, but perceived importance of the system was high among health providers. The ineffectiveness of the integrated system was attributed to policy on the environment, logistical constraints, and orthodox practitioners’ distrust in TMPs.
Madiba, 2010 [20]	Botswana: Tutume	60 orthodox medicine practitioners	Quantitative	Cross-sectional	Determined biomedical health practitioners’ views on collaboration with TMPs.	Convenience sampling	Findings of the study disclosed that collaboration is skewed. The progress of collaboration is thwarted by the absence of specific guidelines on integration.
Maleluka and Ngoepe [27]	South Africa: Limpopo province	27 traditional healers	Qualitative	Hermeneutic phenomenology	Developed a framework to integrate knowledge of traditional healing into the mainstream healthcare system.	Not stated	Demeaning TM practice by not providing adequate support undermined the effectiveness of the integrated system.
Nemutandani et al. [26]	South Africa: Vhembe district, Limpopo Province	77 orthodox medicine practitioners	Qualitative	Not stated	Assessed the perception and experiences of allopathic health practitioners on collaboration with TMPs in the new Democratic South Africa.	Purposive sampling	Findings of the study reported that allopathic practitioners thought of collaboration as compromising the health of service users. An integrated health system in South Africa is not efficient because modern health providers look down on TMPs.

Faculty of Pharmacy (FOP), Ghana Federation of Traditional Medicine Practitioners (GFTMP), Medical Herbalist Association (MHA), Hospital Management (HM), and Pharmaceutical Directorate of Ministry of Health (PDMH).

**Table 2:** Studies on the effectiveness of integrated health systems in Africa.

Reference	Country	Integration Interventions	Year of Inception	Total Years of Int.	Level of Awareness	Level of Usage	Level of Satisfaction	Level of Acceptance	Representative Quotes
Boateng et al. [22]	Ghana	In the year 2001, a council was instituted in Ghana to standardize the practice of TM in the country. In order to legalize this, a policy on the practice of TM was entrenched in 2005 to that effect.	2001	19	Patients at the orthodox medicine unit were not aware of the TM clinic in the hospital. Participants viewed the integrated health system in diverse ways.	Level of usage was low because there was no policy to regulate and publicize the integrated system.	Level of satisfaction among participants was varied, as views reported were contradictory, but perceived satisfaction was high.	Participants stated that the integrated system will be more acceptable if modern medical technologies such as research into efficacy, dosage, standardization, and laboratory services are properly introduced in TM practice.	“*Not aware of operation of TM clinic. Some ailments require TM*” (Client 2, orthodox unit); “*Frequently, we become confused collaboration and integration. It is not entirely integrated*” (key informant); “*The flow of information is good. We have the prescribers’ assembly. They agree on what to be done. They make delivery on what has happened*” (OMP 2).
Agbor and Naidoo [29]	Cameroon	In 1981, Cameroon formally recognised and integrated TM into the health system, but the recognition was not controlled by the MOH. In July 1995, a governmental declaration with the registration number (95-040) mandated traditional practitioners in Cameroon to form local and national associations to regulate the practice of TM.	1981	39	Patients perceive the integrated system as an orthodox health system tolerating traditional care. Therefore, knowledge on integration was low.	Usage of the integrated system is low because of inadequate scientific evaluation problems in TM practice.	Perceived satisfaction was high, but actual satisfaction was low, because the level of integration was low.	Low acceptance of practitioners within the system was because 71% had no professional education as most were trained through apprenticeships and only had primary education.	
Agyei-Baffour et al. [17]	Ghana	Formulation of policy on TM practice in 2005. Creation of the TM Practice Council (TMPC) in 2007 and official TM integrated in 2012.	2005	15	42.2% of participants were aware of the existence of the herbal medicine unit in the study settings.	Usage was moderate, since 42.2% of participants used TM within the health facilities. Additionally, 13% believed that a positive recommendation of TM within the integrated system may increase usage of the system.	Participants were satisfied with the integrated system, as 53% of the respondents indicated that their preferences for TM had increased due to its operation within a hospital setting.	Acceptance of the integrated system was higher among participants with a high socio-economic status than those reported to have a low income.	
Ahenkan et al. [24]	Ghana	The passing of the TM Act in 2000.	2000	20	Awareness of regulations governing TM practice was low as participants, especially TMPs, reported not having knowledge about rules and ethics governing their practice.	Level of usage was reported to be low, particularly among orthodox medicine practitioners, since they were unhappy about people seeking medical care from TMPs. OMPs were unprepared to prescribe TM to clients.	Scarcity of trained TMPs was reported among participants, which was seen to be a disincentive to the integrated system.	Level of acceptance was low among orthodox medicine practitioners due to the exclusion of TM in the medical school curriculum. However, acceptance was high among TMPs due to their positive attitude towards integration.	“*I feel bad, especially when you have the ability to treat a disease but the client kind of seeks care from TMPs*” (medical doctor) “*Who will prescribe TM even if they are added in national drug list? If we’re to prescribe them, then they must be included in what we studied at medical school*.” (medical doctor) “*Because I don’t have equipment to diagnose to know what really causes an ailment, normally I tell my patients to first go to the clinic to get examined, it makes my work easier*.” (TMP)
Appiah et al. [23]	Ghana	Inauguration of Bachelor of Science degree (BSc) in TM at Kwame Nkrumah University of Science and Technology (KNUST) in 2001. Creation of the TM Directorate in Ghana’s Ministry of Health in 2001. Health policy on the safe practice of TM in 2005. Establishment of TM units in selected hospitals in Ghana.	2001	19	Participants were aware of the existence of an integrated system and perceived it to be a step in the right direction, but they reported that administration of the integration procedure needed to be intensified.	Usage of the integrated health system was reported to be low since the national health cover did not embrace TM products and services.	Participants were displeased at the pace of the integration process. Satisfaction was low because participants felt the integrated system should be more efficient than it was at the time of the survey.	Acceptance of the integrated system was high among participants. This was because they recommended that the number of health facilities with TM units should be increased.	“*There’s a need to introduce a good legislative procedure*.” (Participant 2) “*The government should accelerate TM units so that teaching, municipal and district health centres will have them*” (Participant 7) “*Since the hospital board has not defined the exact role the TM unit are to play, TM services are financed by clients through cash and carry system*”. (Participant 11)
Awodele et al. [30]	Nigeria	In 1999, during an ECOWAS special health conference in Abuja Nigeria, the President of the Federal Republic put forth the TM development program and urged for its integration into the formal health system plan.	1999	21	Awareness was low because an intervention put in place to aid collaboration was not well-known among participants.	The lack of regulatory protocol to push the integration agenda has contributed to minimal usage of the system.	Low level of satisfaction due to inadequate support from policy makers.	64% of the participants were willing to succumb to regulations governing the integration process. This depicted a high acceptance among participants.	
Campbell-Hall et al. [25]	South Africa	Approval of the Traditional Health Practitioner Bill	2003	17	Participants were aware of the existence of the integrated health system.	Usage of the system was moderate since participants were aware of the existence of the system and accessed both concurrently or one at a time.	Level of satisfaction was low. Client felt services offered by TMPs are not effective. OMPs also thought that the services offered by TMPs were interfering with their activities. A communication barrier was reported as a challenge to integration.	Acceptance was low among OMPs because they felt clients accessing both health care causes a challenge in the management of infirmities. TMPs were marginalized in the system, but they were willing to learn improved ways of offering care to clients.	“*We started by accessing OM, then proceeded to TMP but utilising TM made the condition worse so stopped and returned to OM.*” (client) “*There is a rift in terms of coordination, interpretation, communication and method of offering health care to clients.*” (OMP)
Falisse et al. [28]	Burundi	Burundi rejuvenated TM practice in the 1980s by seeking UNDP’s support. At the same period, TMPs formed organisations and united with the government to create the Centre for Research and Promotion of TM in Burundi (CRPMT). From 2002 to 2004, an Integrative Medicine (IM) entity was created in the Burundi’s Ministry of Health (MOH) and it paved the way to the lawful practice of integrated health care. Finally, a declaration (number: 100/253/2014) made by government was circulated to guide the practice of IM.	2002	18	91% of orthodox practitioners were aware of the integrated system.	Usage of the system was low because of lopsided power dynamics within the integrated health system.	Satisfaction was low due to poor credibility resulting from inadequate measures to get rid of quack TM practitioners and an unfriendly relationship between TMPs and churches.	Level of acceptance was low among orthodox practitioners as only 19% supported formal integration. Acceptance rate among health users was high as 93% supported integration.	“*A few days ago, we had twin brothers in the paediatric unit, their health was not improving and the parents asked whether they could take them to a TMP. Certainly, we disallowed. Hence, they ran away with the children*.” (OMP 2) “*Not long ago, the Catholic Church organised a parade led by a cross of Jesus, the aim was to wipe out healers and their practices. I am surprised. The priest-healer of Bururi, is he not Catholic? And is he not curing people himself?”* (TMP 1) “*Presently, children go to school and they get taught that TM does not heal. And they have a bad image of AM. But they are wrong.*” (TMP 1)
Gyasi et al. [12]	Ghana	The MOH in partnership with the Ghana Federation of TMPs endorsed a strategic plan for the promotion of TM practice (2000–2004). The plan comprised the formation of a comprehensive training program in TM; in line with this strategy, the KNUST is presently running a BSc degree in TM. Again, the Centre for Scientific Research into Plant Medicine has been in operation since 1975 to promote TM practice.	2000	20	Study participants were aware of the existence of integration between the two health systems, but admitted that the system was not effective.	Patronage of integrated health services was low because of a weak referral system.	Satisfaction was low among orthodox practitioners due to inadequate credibility backing TM practice, whereas health care users were satisfied with the system.	Acceptance was high among health care users, but unpopular among TMPs and orthodox medicine practitioners.	*“When I told a midwife that I have utilised TM to ease my morning sickness like extreme vomiting, she was upset with me, and ordered me to go and access care somewhere else. You see! One nurse also blamed me of not acting properly having used TM, meanwhile the TM was efficient.”* (Health user 1) *“I don’t refer my clients to the traditional healers and I don’t think it’s the right thing to do at the moment. This is because I can’t guarantee the quality of care they (the healers) will provide to the poor and the helpless patients. I know most of the healers depend on spirits, deities and witchcrafts which cannot be explained in the medical language I understand. If I don’t understand and also can’t be sure of the treatment outcomes of the patients, then there is no need to refer them to see a traditional healer for their problems.”* (Health professional 1)
Kaboru et al. [31]	Zambia	Institution of the Traditional Health Practitioners Association of Zambia	Not stated	-	Awareness was low, as 24% of participants reported of knowing and having experience with the integrated health system.	Usage was reported to be moderate among TMPs as 53% reported directing their clients to seek orthodox health care. The reverse was reported among OMPs, because only 4% recommended TM to clients.	Perceived satisfaction was high among TMPs since 97% perceived their practice to be important in the health system.	Perceived acceptance was reported to be high among participants as 77% of OMPs and 97% of TMPs thought there was the possibility for OMPs to learn from TMPs. On the other hand, 97% of OMPs and 90% of TMPs reported a likelihood to learn from OMPs.	
Madiba [20]	Botswana	Enactment of the national health policy of Botswana stipulating the nature of integration through common grounds for learning and communication between the two health systems.	1995	25	Level of awareness was low, because merely 18.6% of participants knew about the existence of an integration policy. However, knowledge about client usage of TM was moderate, at 50%.	Usage of the system was recounted to be low since 90% of participants registered their unwillingness to refer clients to TMPs.	Satisfaction was also minimal because 70% of participants were not pleased with TM practice.	Acceptance of the integrated system was low due to skewed power relations, where OMPs felt their role in the system was superior and they were unprepared to welcome TM. About 73% of participants had at no time cooperated with TMPs. Likewise, only 6% of participants perceived TMPs as co-workers.	
Maleluka and Ngoepe [27]	South Africa	The Government of South Africa enacted a law on Traditional Health Practitioners (THP) Act in 2007 to serve as the foundation of TM practice and promote integration. Another measure was the creation of the Institution of Traditional Health Practitioners Council of South Africa (THPCSA) to guide the activities of practitioners.	2007	13	Both practitioners knew of the existence of the integrated system. This was evident in the interaction between healers and orthodox practitioners (training and referrals).	Level of usage was low as participants reported of referring clients to hospitals, but they did not receive referrals from hospitals.	Low satisfaction echoed in the views of participants as they claimed that integration was one-sided.	Level of acceptance was low as TMPs felt marginalized. Again, orthodox practitioners did not accept the activities of the healers.	“*Integration is skewed because health facilities do not refer clients to us but we do refer clients to health centres*.” (Respondent A) “*After referring clients to hospitals nurses sometimes fight with both clients and TMPs enquiring why they (clients) consulted TMPs first instead of going to the hospitals*.” (Respondent C) “*I had a rapport with our local health centre and we had challenges when it came to issues of payment*.” (Respondent E)
Nemutandani et al. [26]	South Africa	Dissemination of Traditional Health Practitioners Act (Number 22)	2007	13	Level of awareness among participants regarding the existence of the Traditional Health Practitioners Act was low.	Usage was low, because TM practice was perceived to compromise the standard of health that should be delivered to clients.	Satisfaction was reported to be low among participants as some were concerned about the level of medical knowledge of TMPs.	Acceptance was also recounted to be low among study participants.	“*Does it mean that TMPs are health employees like us? What is happening? Yet, we were not once asked about the Act. How will it function*?” (Medical services manager 3) “*If TMPs are rightly educated, we will collaborate with them as treatment supporters. Won’t TMPs combine the treatment with medicinal plants?”* (Physician 1)

Int: Integration.

**Table 3:** Quality assessment of included studies using the quality assessment tool for studies with diverse designs (QATSDD).

QATSDD Criteria	1	2	3	4	5	6	7	8	9	10	11	12	13	14	15	16	Total Score	% of Total Score	Grade
Boateng et al. [22]	0	3	3	2	2	3	3	0	N/A	N/A	2	2	2	2	0	2	26/42	62	Good
Agbor and Naidoo [29]	0	3	3	3	2	2	2	2	0	2	N/A	3	2	N/A	0	0	24/42	57	Good
Agyei-Baffour et al. [17]	0	3	3	3	3	3	0	3	0	3	N/A	3	2	N/A	2	2	30/42	71	Good
Ahenkan et al. [24]	3	3	3	3	3	1	3	2	N/A	N/A	3	3	3	3	0	0	33/42	79	Good
Appiah et al. [23]	3	3	3	3	2	3	2	3	N/A	N/A	3	3	3	3	0	2	36/42	86	Excellent
Awodele et al. [30]	0	3	3	3	2	3	0	3	0	2	N/A	3	3	N/A	0	0	25/42	60	Good
Campbell-Hall et al. [25]	0	3	3	2	3	3	1	1	N/A	N/A	3	3	1	3	0	0	26/42	62	Good
Falisse et al. [28]	0	3	3	3	3	3	2	1	0	3	3	3	3	3	1	0	34/48	71	Good
Gyasi et al. [12]	0	3	3	2	2	3	3	2	N/A	N/A	3	2	3	3	1	0	30/42	71	Good
Kaboru et al. [31]	0	3	3	3	3	2	2	3	2	3	N/A	1	2	N/A	0	3	29/42	69	Good
Madiba [20]	0	3	2	1	2	3	1	3	2	1	N/A	3	2	N/A	3	0	26/42	62	Good
Maleluka and Ngoepe [27]	0	3	2	3	3	3	2	3	N/A	N/A	3	2	2	3	0	2	31/42	74	Good
Nemutandani et al. [26]	0	3	3	3	3	3	1	1	N/A	N/A	3	3	0	3	0	3	29/42	69	Good

0 represents ‘criterion not mentioned at all’, 1 represents ‘very slightly mentioned criterion’, 2 represents ‘moderately mentioned criterion’, 3 represents ‘fully explained criterion’, and N/A means ‘criterion not applicable’.

## Supplementary Materials

The following are available online at https://www.mdpi.com/1010-660X/56/6/271/s1, Table S1: Search Strategy for effectiveness of integrated health systems.
