# Supplementary material for: Effectiveness of Integrated Health Systems in Africa: A Systematic Review
Source: Medicina (Kaunas). 2020 May 29;56(6):271. doi: 10.3390/medicina56060271 (PMC7353894; doi:10.3390/medicina56060271)
Supplement: Supplementary file 1 [file medicina-56-00271-s001.pdf]

Supplementary Table 1: Search strategy for effectiveness of integrated health systems

Database	Search terms
Ovid Medline	1. "Delivery of Health Care, Integrated"/<br>2. "Delivery of Health Care"/<br>3. integrated health system*.mp.<br>4. hospitals/ or exp health services/ or community health services/<br>5. ((integrated or holistic) and health and (system* or clinic* or service*)).mp. [mp=title, abstract, original title, name of substance word, subject heading word, floating sub-heading word, keyword heading word, organism supplementary concept word, protocol supplementary concept word, rare disease supplementary concept word, unique identifier, synonyms]<br>6. 1 or 2 or 3 or 4 or 5<br>7. exp medicine, traditional/ or spiritual therapies/ or faith healing/ or medicine, african traditional/<br>8. (alternative medicine* or alternative therap* or complementary medicine* or complementary therap or "faith healing" or "spiritual therap*" or "spiritual medicine*" or "traditional medicine*" or "indigenous medicine*" or "native medicine*" or "herbal medicine*").mp.<br>9. 7 or 8<br>10. 6 and 9<br>11. 1 or 2 or 3 or 5<br>12. 9 and 11
Scopus	((TITLE-ABS-KEY (integrated OR holistic) AND TITLE-ABS-KEY (health) AND TITLE-ABS-KEY (system* OR clinic* OR service*))) AND ((TITLE-ABS-KEY ("alternative medicine*" OR "alternative therap*" OR "complementary medicine*" OR "complementary therap*" OR "faith healing" OR "spiritual therap*" OR "spiritual medicine*" OR "traditional medicine*" OR "indigenous medicine*" OR "native medicine*") OR TITLE-ABS-KEY ("herbal medicine*")))
Emcare	1. integrated health care system/<br>2. (alternative medicine* or alternative therap* or complementary medicine* or complementary therap or "faith healing" or "spiritual therap*" or "spiritual medicine*" or "traditional medicine*" or "indigenous medicine*" or "native medicine*" or "herbal medicine*" or "integrative medicine").mp. [mp=title, abstract, heading word, drug trade name, original title, device manufacturer, drug manufacturer, device trade name, keyword]<br>3. traditional medicine/ or african medicine/ or herbal medicine/<br>4. alternative medicine/ or spiritual healing/<br>5. integrative medicine/<br>6. 2 or 3 or 4 or 5<br>7. ((integrated or holistic) and health and (system* or clinic* or service*)).mp. [mp=title, abstract, heading word, drug trade

	name, original title, device manufacturer, drug manufacturer, device trade name, keyword]<br>8. 1 or 7<br>9. 6 and 8
CINAHL	1 (MH "Health Care Delivery, Integrated") OR (MH "Systems Integration")<br>2 ((integrated or holistic) and health and (system* or clinic* or service*))<br>3 1 OR 2<br>4 (MH "Medicine, Herbal") OR (MH "Spiritual Healing") OR (MH "Medicine, Traditional") OR (MH "Medicine, African Traditional") OR (MH "Integrative Medicine")<br>5 (alternative medicine* or alternative therap* or complementary medicine* or complementary therap or "faith healing" or "spiritual therap*" or "spiritual medicine*" or "traditional medicine*" or "indigenous medicine*" or "native medicine*" or "herbal medicine*" or "integrative medicine")<br>6 4 OR 5<br>7 3 AND 6
Web of Science	**Topic:** (integrated OR holistic) *AND* **TOPIC:** (health) *AND* **TOPIC:** (system* OR clinic* OR service*) *AND* **TOPIC:** ("alternative medicine*" OR "alternative therap*" OR "complementary medicine*" OR "complementary therap*" OR "faith healing" OR "spiritual therap*" OR "spiritual medicine*" OR "traditional medicine*" OR "indigenous medicine*" OR "native medicine*" OR "herbal medicine*") **Timespan:** All years. **Indexes:** SCI-EXPANDED, SSCI, A&HCI, CPCI-S, CPCI-SSH, ESCI, CCR-EXPANDED, IC**...**
Google Scholar	("Traditional medicine" OR "herbal medicine" OR "alternative medicine" OR "indigenous medicine" OR "complementary medicine") AND integrated
